# Technical and financial feasibility of a chemicals recovery and energy and water production from a dairy wastewater treatment plant

**DOI:** 10.1038/s41598-024-61699-8

**Published:** 2024-05-15

**Authors:** Ali Basem, Dheyaa J. Jasim, Pooya Ghodratallah, S. AbdulAmeer, Ahmed Mohammed Mahmood, Wisam J. Khudhayer, Hasan Khalid Dabis, Mohammad Marefati

**Affiliations:** 1 Faculty of Engineering, Warith Al-Anbiyaa University, Karbala, 56001 Iraq; 2 Department of Petroleum Engineering, Al-Amarah University College, Maysan, Iraq; 3 Department of Civil Engineering, College of Engineering, Cihan University-Erbil, Erbil, Iraq; 4 Faculty of Engineering and Natural Sciences, Istanbul Okan University, Istanbul, Turkey; 5 Department of Automobile Engineering, College of Engineering, Al-Musayab, University of Babylon, Babylon, Iraq; 6 Department of Optical Techniques, Al-Noor University College, Mosul, Iraq; 7 Department of Energy Engineering, College of Engineering, Al-Musayab, University of Babylon, Babylon, Iraq; 8 Ahl Al Bayt University, Karbala, Iraq; 9 Department of Energy Engineering, Faculty of Natural Resources and Environment, Science and Research Branch, Islamic Azad University, Tehran, Iran

**Keywords:** Chemicals recovery, Energy and water production, Dairy wastewater treatment plant, Technical assessment, Financial feasibility, Permeate water flux, Chemistry, Energy science and technology

## Abstract

Due to the high volume of wastewater produced from dairy factories, it is necessary to integrate a water recovery process with the treatment plant. Today, bipolar membrane electrodialysis units (BMEUs) are increasingly developed for wastewater treatment and reutilizing. This article aims to develop and evaluate (technical and cost analyses) a combined BMEU/batch reverse osmosis unit (BROU) process for the recovery of chemicals and water from the dairy wastewater plant. The combined BROU/BMEU process is able to simultaneously produce water and strong base-acid, and reduce power consumption due to the injection of concentrated feed flow into the BMEU. A comprehensive comparative analysis on the performances of two combined and stand-alone BMEU configurations are developed. The proposed combined technology for dairy factory wastewater treatment is designed on a new structure and configuration that can address superior cost analysis compared to similar technologies. Further, the optimal values of permeate flux and current density as two vital and influencing parameters on the performance of the studied dairy wastewater treatment process were calculated and discussed. From the outcomes, the total cost of production in the combined configuration has been reduced by approximately 26% compared to the stand-alone configuration. Increasing the feed concentration rate using the batch reverse osmosis process for the dairy wastewater treatment process can be an ideal solution from an economic point of view. Moreover, point (current density, feed concentration rate, total unit cost) = $\left(328.9, 7, 14.37\right)$ can be considered as an optimal point for the economic performance of the studied wastewater treatment process.

## Introduction

Nowadays, the freshwater resources scarcity has become a serious challenge all over the world as to the rapid increase in demands due to the increasing industrial, agricultural, economic, and social developments^1,2^. On the other hand, in addition to increasing environmental crises, the discharge of industrial wastewater seriously aggravates the water scarcity problem by contaminating surface and underground water^3,4^. In the meantime, the industrialization growth increases the discharge rate of high-salinity wastewater. In such a situation, water cannot be directly utilized for potable, agricultural, and industrial purposes^5,6^. Accordingly, the increasing development of wastewater treatment and resource recycling technologies is necessary to reduce the environmental impacts of wastewater discharge^7–9^. Among the various industries, the dairy factory is one of the industries that produces huge wastewater; such that to produce one liter of milk, around 2.5 L of water is consumed and the same amount of wastewater is produced^10,11^. Wastewater treatment and recycling in such industries can simultaneously reduce freshwater consumption and the environmental impacts of wastewater discharge into the environment^12,13^. The wastewater from a dairy factory is mainly comprised of inorganic salts, casein, disinfectants, sodium (from caustic soda), detergents, etc.^14,15^.

It was reported that, nearly 60% of the total water consumption of a dairy factory is related to the on-site cleaning process, and a high share of wastewater from dairy factories is related to this process^16^. All initial, intermediate, and final water rinse steps, as well as alkaline and acid washes, are related to the on-site cleaning process. For this purpose, compounds such as potassium hydroxide, sulphuric acid, peracetic acid, sodium hydroxide, hydrochloric acid, nitric acid, and phosphoric acid are utilized in dairy factories. All these compounds are generally present in the wastewater discharged from the factory^17^. Therefore, the treatment and recycle of wastewater from dairy factories with this volume of produced waste can be fruitful in order to achieve their sustainable development^18^. In addition, reuse of recovered water and chemicals in the same plant can address a profitable route for the industry.

A membrane filtration process is a technology to separate the cleaning solution and the contaminant at the end of the on-site cleaning process, which is capable of reproducing a diluted alkaline solution^19^. Ultra-filtration, nano-filtration, electrodialysis, micro-filtration, BMEU, etc. are among such common membrane technologies for material recovery^20^. It was reported that using a nano-filtration membrane approach to treat wastewater from on-site washing processes could achieve superior separation behavior (eliminating the chemical oxygen demand up to 75% and completely removing suspended solids)^21,22^. Kowalska^23^ evaluated the unit and integrated pressure-driven nano-filtration and ultra-filtration membrane processes for the purification and concentration of contaminated cleaning solutions in the dairy industry. He reported that the integrated separation approach could provide high separation rates with a slight weakening of the permeate detergency properties. However, filtration membranes require maintenance and direct monitoring after each cycle. In addition, achieving high rate chemical recovery needs the addition of acidic reagents. Indeed, in order to obtain a diluted cleaning solution, cleaning chemicals can be added and/or the reverse osmosis process can be employed^24^.

The employ of reverse osmosis process for re-concentration in wastewater treatment process had been reported in some publications. Marotta Alfaia et al.^19^ developed the impacts of landfill leachate pre-treatment process on the nano-filtration and reverse osmosis membranes considering the effects of different parameters (such as permeate water flux, quality of leachate, and fouling resistance). They reported that the nano-filtration and reverse osmosis processes were not suitable as a stand-alone stage in a landfill treatment cycle, and a pre-treatment process was suggested. C. Bortoluzzi et al.^20^ reported that an integrated process based on nano-filtration, micro-filtration, and reverse osmosis was able to treat dairy factory wastewater. However, this process required very high pressure and resulted in the production of organic sediment. Chen et al.^25^ developed a two-phase dye wastewater treatment process under a reverse osmosis process and non-thermal plasma degradation. That work could enhance the energy yield of the plasma-based dye wastewater treatment process by improving mass transfer. Yu Chen et al.^26^ reported that Ammonium bicarbonate could improve the performance of the osmotic photo-bioreactor technology in both water treatment and carbon neutralization. Hu et al.^27^ evaluated the behavior of a reverse osmosis membrane under the osmotic cleaning of typical inorganic and organic foulants for a textile printing and dyeing wastewater treatment process. The results revealed that the osmotic cleaning process could extend the water flux recovery capability by 95% in a relatively long period of time in addition to providing high performance. An et al.^28^ developed the filtration performance and energy consumption for reverse osmosis process to treat semiconductor wastewater. They found that for the advanced oxidation process scanning electron microscopy with ozone, the oxidation effect of organic matter was insignificant. Moreover, among the three applied reverse osmosis membranes, ESPA was suitable in terms of process stability. Alalam et al.^29^ developed an adapted reverse osmosis treatment process to reduce the environmental impacts of white wastewaters for a dairy effluent. Lakra et al.^30^ reported that lipids, protein, and carbohydrate separation from dairy wastewater could lead to economic benefits. The proposed process was based on forward osmosis and ultra-filtration processes. However, those processes were pressure driven and energy-intensive technologies, and the presence of the aforementioned compounds caused fouling. Ju Kim et al.^16^ integrated the nano-filtration and forward osmosis processes for recovery of cleaning agents from on-site cleaning dairy wastewater. Form the results, the nano-filtration steps were found to eliminate remarkable amounts of lactose and protein contents. In addition, thin-film composite membrane was preferred than the cellulose triacetate membrane due to enhance water permeability. Further, the recovered cleaning agents exhibited cleaning yield comparable to fresh cleaning agents.

Therefore, the stand-alone use of membrane process for wastewater treatment can cause the formation of heavy membrane sediment and/or decrease membrane water permeability^31^. Accordingly, the membrane application can be a modern and appropriate option if integrated with the wastewater treatment process in order to recover water^32^. These goals (reproduction of chemicals and simultaneous recovery of water) cannot be achieved through filtration and reverse osmosis processes, and the design of an advanced combined system is necessary^33^. BMEU is an advanced and compact electrochemical technology capable of producing base and acid through the separation of water and base-acid from salt solutions. This process is carried out via the ions movement through the membrane and by applying voltage. It has a simpler structure and operation compared to traditional processes^34^. It was reported that the use of BMEU to produce base and acid from Sodium chloride solution could achieve both low and high concentrations^35^. In some publications, the combination of reverse osmosis and electrodialysis processes for base and acid production was reported. Liu et al.^36^ developed the application of the BMEU in electrodialysis desalination considering the effects of operating parameters. They found that the BMEU could be a sustainable and safe approach to energy conversion and storage. Chen et al.^37^ reported that BMEU could use and convert the dissolved waste salt into valuable base and acid simultaneously. Further، the in-situ BMEU could offer significant environmental and cost advantages. Abusultan et al.^38^ developed a re-mineralization process for reverse osmosis permeate based on a combined ion exchange-bipolar membrane hybrid process. The preparative ion exchange chromatography was employed to separate the cations. Further, the elution process could determine yield and purity of the bivalent ion recovery. The cation stream could be utilized to remineralize reverse osmosis permeate for freshwater.

Jiang et al.^39^ proposed a hybrid zero-liquid discharge of reverse osmosis, electrodialysis, and BMEU for salt recovery and base-acid generation under the cold-rolling wastewater treatment process. They determined the appropriate membrane type, voltage drop, and volume ratio. They found that the hybrid process could offer potential applicability for the sustainable treatment of cold-rolling wastewater. Yang et al.^40^ employed the BMEU technology for separate inorganic salts from real landfill leachate reverse osmosis concentrate and reclaiming inorganic acids and bases. They considered the effects of membrane surface flow velocity, working voltage, and base-acid concentration. Their work was based on a new strategy to realize the sustainable use of inorganic salts from the landfill leachate reverse osmosis concentrate. Tang et al.^41^ employed BMEU to treat the concentrated brine obtained during reverse osmosis process for recover valuable resources and generate base-acid considering the impacts of working voltage, low velocity, feed concentration ratio, and unusual phenomena. The results indicated that the cost of the BMEU was around 10.54 YCH/kg NaOH (Sodium hydroxide). Zhang et al.^42^ developed specific protocols to establish a fruitful pathway to develop advanced membrane processes for salinity gradient energy harvesting and based-acid generation under a BMEU technology. The potential of circular economy and water management through the development of the BMEU technology in order to recover valuable products and produce base-acid was reported by Gonzalez et al.^43^. Cassaro et al.^44^ designed a BMEU process with a membrane area of around 19.2 m^2^ to produce base-acid from a saline waste stream discharged from a reverse osmosis-seawater desalination process. They reported that designing and achieving an optimal configuration was the main step to commercialize BMEU and the competitive market.

Industrial wastewater treatment can jointly address energy and environmental issues. Based on a comprehensive survey, due to the high volume of wastewater produced from dairy factories, it is necessary to integrate a water recovery process with the treatment plant. However, very rare works were reported on the application of electrodialysis process with bipolar membrane for the treatment of dairy wastewater. In addition, most of the studies had focused on the technical analysis of the wastewater treatment process, and less had been discussed about the economic feasibility of wastewater treatment and the recovery of chemicals and water. Accordingly, this article aims to develop and evaluate a combined BMEU/ BROU process for the recovery of chemicals and water from the dairy wastewater plant. In the proposed combined process, it is possible to simultaneously produce water and base-acid from the final dairy effluent. The technical and cost analyses of the combined wastewater treatment process are comprehensively discussed and investigated. In addition, a comprehensive comparative analysis on the performances of two combined and stand-alone BMEU configurations are developed. Further, the behaviors of the configurations under different parameters (such as feed concentration rate and current density) have been investigated. The proposed combined technology for dairy factory wastewater treatment is designed on a new structure and configuration that can address superior cost analysis compared to similar technologies. The summary of contributions and aims of the article are as follows:

- The design of the combined BROU/BMEU process is able to produce strong base-acid and reduce power consumption due to the injection of concentrated feed flow into the electrodialysis process with bipolar membrane;
- Using BROU instead of the conventional reverse osmosis process can bring lower energy consumption (especially at high recovery rates);
- Calculation and discussion of the optimal values of permeate flux and current density as two vital and influencing parameters on the performance of the studied dairy wastewater treatment process;
- Due to the reutilize of wastewater treatment products in the dairy plant, the proposed plan can deal with the concept of circular economy and wastewater management;
- Addressing a combined structure and configuration for dairy wastewater treatment that can offer superior economic performance compared to similar technologies.

## System description

**Figure 1:**
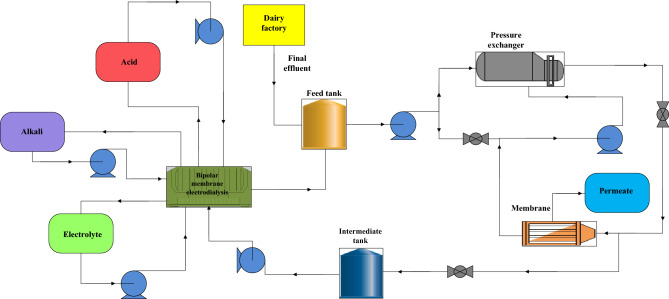
Proposed schematic for the combined wastewater treatment process.

Industrial wastewater treatment can jointly address energy and environmental issues. In this article, a combined BMEU/BROU process for the recovery of chemicals and water from the wastewater of an industrial process (dairy production) is developed and evaluated. The considered schematic for the combined wastewater treatment process is demonstrated in Fig. 1. As can be seen, the proposed wastewater treatment process is comprised of two BMEU and BROU processes. In this combined process, in addition to producing water from the final effluent of dairy (by using BROU process) base and acid can also be produced. Indeed, first water is recovered through BROU and then base and acid are produced through BMEU and the concentrated reject flow. The outlet stream of BMEU process is added to the inlet stream (feed tank) for reuse after adjusting the concentration. It is assumed that the acid and base of BMEU can meet the quality requirements. The performance of the studied combined wastewater treatment process is also compared with the behavior of a stand-alone BMEU process. In a stand-alone BMEU process, the treated effluent is directly directed to the BMEU to carry out the acid and base production process. During this process, by removing the dissolved ions, the feed flow turns into a diluted salt solution. For both combined and stand-alone configurations, the diluted stream concentration is assumed to be the same.

During the electrodialysis process with a bipolar membrane, water is directed from the feeding side to the base and acidic flows through the ion exchange membrane. Water transfer operation is done due to osmotic pressure difference. The batch reverse osmosis process is a cyclic process; each cycle is based on two phases of pressurization and purge-refill. In these phases, permeate water and reject flow are produced, respectively. During the pressurization mode, the piston is directed to the right by feeding water to the pressure exchanger. The water is forced out from the other side of the piston, which is mixed with the recirculation flow. Then, it directed to the feed side of the reverse osmosis module through the main valve. The reject flow of the module is recirculated through a recirculation pump. During this stage permeate is achieved and continues until the piston reaches the right-side end. In this cycle, the pressure and water concentration (in the recirculation loop) gradually raise. The second operational phase of the BROU (i.e., purge-refill) is based on removing very concentrated saltwater from the module vessel, which must be replaced with feed water. During this stage, the purge valve is open. Besides that, the bypass valve is also open in order to divert the fresh directed feed water to the exchanger left. Finally, the recirculation pump drives the piston to the initial position. Therefore, the operation of the studied combined wastewater treatment process (BROU/BMEU) can be summarized as follows:

- Separation of dissolved salt and water under the dairy plant wastewater treatment process through BROU;
- Concentrating the reject flow of the BROU with dissolved salt and directing the mixture to the inlet of the BMEU. Indeed, using BROU before BMEU to concentrate the feed flow;
- Concentrated feed flow is able to produce strong base-acid and reduce power consumption.

As mentioned, the wastewater treatment process in the current research is based on dairy wastewater. The quality of effluent from dairy plants mainly depends on processing parameters (e.g., design, production and operational methods, type of product, etc.) and water management programs^45^. Based on this, the wastewater from dairy plants is based on three major categories^46^: (1) Water utilized in the cooling/heating heat exchangers (due to less pollution, it can be reutilized and/or discharged into the environment without serious concern), (2) Wastewater related to sanitary purposes, and (3) Wastewater related to the process of cleaning different dairy components.

The characteristics of dairy effluent considered for the present research are given in Table 1^47–49^, which mainly comprises total dissolved solids (TDS), total suspended solids (TSS), chemical oxygen demand (COD), and biological oxygen demand (BOD). It is assumed that an average amount of ~ 2200 mg/L of dissolved solids are available in the wastewater, as reported in^50^. It should be noted that the presence of dissolved solids is due to acid and alkaline wash during the Cleaning-in-place process. The wastewater treatment plant is based on biological treatment process (membrane bioreactor), which can significantly reduce suspended solids and organic matter. Moreover, hydrochloric acid (HCl) and NaOH were considered as acid and base products of the on-site cleaning process. It should be emphasized that even though dairy wastewater is treated by an Effluent Treatment Plant (ETP), there may still be a small quantity of organic and suspended particles present in the wastewater that could necessitate pretreatment. Nevertheless, a study on the BMEU treating an industrial NaCl stream with some organic matter present revealed no biofouling on the membrane and reduced contamination levels after extended recirculation^51^. Finally, although the proposed wastewater treatment process may require a pre-treatment process, due to the lower participation of this process in the total cost, its calculations are not considered.

**Table 1:** The characteristics of dairy effluent considered for the present research, the values are average data reported in the literature^50,52–60^.

Characterization data	Raw wastewater (mg/L)	Treated effluent (mg/L)
Total dissolved solids	2210	1216
Total suspended solids	553	37
Chemical oxygen demand	2120	124
Biological oxygen demand	732	12

Note that, since the current research is based on a numerical analysis (and is not experimental in nature) and the evaluation is based on a simulation developed under a mathematical analysis, therefore, data reported in similar literature have been used to collect data. Indeed, the values presented in Table 1 are the average data of dairy wastewater characteristics reported in the literature^50,52–60^. However, the developed simulation and analysis has been done in a way that can be easily generalized for other similar cases.

## System modelling

### Technical analysis

**Figure 2:**
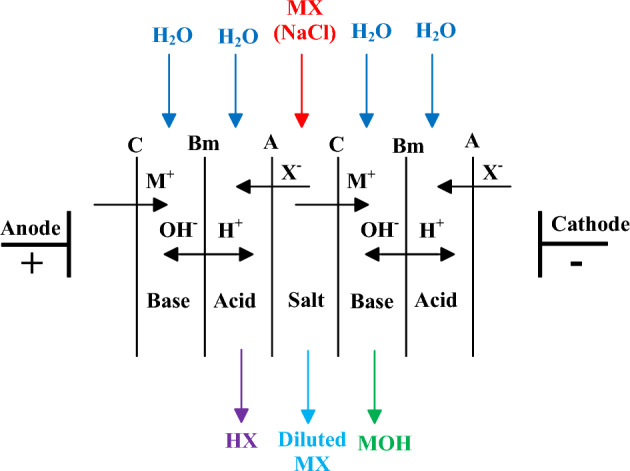
Structure of the electrodialysis process with bipolar membranes (adapted from^64^).

The electrodialysis process with bipolar membranes is employed to separate salt by splitting of electrodialysis water to produce bases and acids. This process occurs through the applying electric power. The bipolar membranes enhance the water splitting into hydroxide ions and protons^61^. As shown in Fig. 2, the electrodialysis process with bipolar membranes is comprised of anion, bipolar, and cation exchange membranes that are connected in parallel. This connection leads to the formation of a single cell between two electrodes. The whole process done in the BMEU to produce base and acid from salt solution is as follows: by applying an electric current, the anions and cations of the inlet salt solution (to the middle compartment) are directed to the anode and cathode electrodes, sequentially^62^. Afterwards, the ions pass through the exchange membranes and are directed to the adjacent compartment. In this compartment, protons and hydroxide ions are produced through the bipolar membrane. Finally, the productions of base and acid are done through the combination of cations and anions with hydroxide ions and protons, sequentially. According to the publications, the performance of the BMEU is highly dependent on the bipolar membrane performance^63^.

The stack structure of a BMEU is basically similar to the stack structure of conventional electrodialysis (sheet flow stack) and the only difference is the triplet-cell structure. This means feeding three separate streams through the manifold in the separate cells to create a duplicate unit. The necessary membrane area, which is defined as the unit cell area (triplet-cell structure), is formulated by the following equation^64^:

1 $${A_{BMEU}} = \frac{{F \times {Q_f}}}{{j \times {U_I}}} \times \left( {C{P_{s,in}} - C{P_{s,out}}} \right)$$

where, $F$, ${Q}_{f}$, $j$, ${U}_{I}$, and ${CP}_{s}$ are the Faraday constant, inlet feed flow rate, current density, current utilization, and salt stream's concentration, respectively. In addition, the area of the cell unit is a function of the electric current passing through BMEU, which is expressed as follows:

2 $${I_{BMEU}} = {A_{BMEU}} \times j$$

The length of the membrane stack is a function of the area and current of the membrane, which is expressed by^64^:

3 $${L_{mem}} = \frac{{{A_{BMEU}}}}{{{N_{cell,BMEU}} \times {W_{cell}}}}$$

where, ${N}_{cell,BMEU}$ and ${W}_{cell}$ are the cell pair numbers and cell width, respectively. Further, the mean salt concentration (${CP}_{s,avg}$) for an ion is defined as the integral average along the length of the path^50^:

4 $$C{P_{s,avg}} = {\left( {Ln \, \left( {\frac{{C{P_{s,in}}}}{{C{P_{s,out}}}}} \right)} \right)^{ - 1}} \times \left( {C{P_{s,in}} - C{P_{s,out}}} \right)$$

As mentioned, electrodialysis process with bipolar membrane consumes electricity. Accordingly, the specific electricity consumption of this process can be estimated by the following equation^38^:

5 $${\overline P_{BMEU}} = \frac{{\Delta {V_{cell}} \times {I_{BMEU}}}}{{Q_f}}$$

where, ${\Delta V}_{cell}$ is the cell voltage loss. The total voltage drop in the mentioned process can be considered as a sum of the voltage losses caused by the membranes and the solution resistances and the potential of the water dissociation. Considering some assumptions (see Table 6), the voltage drop of the cell can be formulated as follows^64^:

6 $$\left\{ \begin{gathered} \Delta {V_{cell}} = \frac{RT}{F}.\ln \left( {\frac{{C{P_{tr,{H^+ }}}.C{P_{tr,O{H^- }}}}}{{C{P_{a,{H^+ }}}.C{P_{b,O{H^- }}}}}} \right) + j \times \left[ {\varepsilon .x + A{R_{tr}} + A{R_{amem}} + A{R_{bm}} + A{R_{cmem}}} \right] \hfill \\ {\text{where,}} x = \frac{{\ln \left( {\frac{{C{P_{s,in}}}}{{C{P_{s,out}}}}} \right)}}{{{H_s}.\left( {C{P_{s,in}} - C{P_{s,out}}} \right)}} + \frac{{\ln \left( {\frac{{C{P_{a,in}}}}{{C{P_{a,out}}}}} \right)}}{{{H_a}.\left( {C{P_{a,in}} - C{P_{a,out}}} \right)}} + \frac{{\ln \left( {\frac{{C{P_{b,in}}}}{{C{P_{b,out}}}}} \right)}}{{{H_b}.\left( {C{P_{b,in}} - C{P_{b,out}}} \right)}} \hfill \\ \end{gathered} \right.$$

where, $R$, $T$, $\varepsilon$, $AR$, and $H$ are the gas constant, the absolute temperature, cell thickness, area resistance, and equivalent conductivity, respectively. Further, the subscripts $tr$, $amem$, $a$, $bm$, $b$, and $cmem$ refer to the transition region, anion-exchange membrane, acid, bipolar membrane, base, cation-exchange membrane, respectively. The equivalent conductivity values for different solutions can be determined based on the Equations stated in Table 2.

**Table 2:** Equations for calculating the equivalent conductivity values for different solutions ^50,64^.

Solution	Range	Equation no.	Equation
NaCl solution	$C{P_{s,avg}} > 1{\text{ mol/L}}$	Equation (7)	${H_s} = 9.19719 + C{P_{s,avg}} \times \left( { - 0.862905} \right)$
	$C{P_{s,avg}} \leqslant 1{\text{ mol/L}}$	Equation (8)	${H_s} = 13.04924 + CP_{s,avg}^{0.27816} \times \left( { - 4.47748} \right)$
NaOH solution	$C{P_{b,avg}} \leqslant 1.5{\text{ mol/L}}$	Equation (9)	${H_b} = 24.59432 + CP_{b,avg}^{0.47072} \times \left( { - 6.92179} \right)$
HCl solution	$C{P_{a,avg}} > 1{\text{ mol/L}}$	Equation (10)	${H_a} = 36.6063 + C{P_{a,avg}} \times \left( { - 4.17} \right)$
	$C{P_{b,avg}} \leqslant 1{\text{ mol/L}}$	Equation (11)	${H_a} = 42.443 + CP_{a,avg}^{0.43556} \times \left( { - 8.83285} \right)$

**Figure 3:**
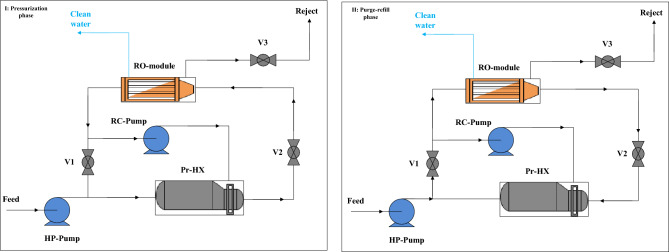
Structure of the batch reverse osmosis process based on the two operational pressurization and purge-refill phases (V1: Bypass valve, V2: main valve, V3: purge-refill valve), adapted from^66^.

BROU is another unit considered in the proposed wastewater treatment process. The batch reverse osmosis process is a cyclic process; each cycle is based on two phases of pressurization and purge-refill. In these phases, permeate water and reject flow are produced, respectively^65^. The structure of the batch reverse osmosis process based on the mentioned two operational phases is depicted in Fig. 3. The design of BROU based on a cyclic process (i.e., in this process, the pressure reaches from the minimum value to the peak value) can minimize the average inlet pressure in the desalination process and consequently the specific energy consumption. It can be seen from Fig. 3 that a BROU is comprised of the normal reverse osmosis housing, the housing as a pressure exchanger, a feeding pump, a recirculating pump, and three valves. The peak pressures in the BROU and conventional reverse osmosis processes (under a continuous process) is considered similar, the only difference being the momentarily achieved pressure. At other times of the cycle, the BROU pressure is less than the pressure of the conventional reverse osmosis process^66^. At higher recovery ratios, the energy saved in the BROU is greater than in a conventional reverse osmosis process (due to lower average pressure requirements). Accordingly, due to the reduction of energy consumption at high recovery scales, a BROU can be more beneficial than the conventional reverse osmosis process^67^.

In this article, BROU is designed based on a free piston in order to concentrate the inlet feed flow in wastewater treatment. In addition, the membrane module is of Dupont/BW30/365 type. Further, the pressure exchanger and the membrane housing are of the Codeline Pentair/80E30 type^66^. Accordingly, the total volume of the process that occurred in the BROU can be expressed as follows^66^:

12 $$\begin{gathered} {V_{BROU,tot}} = {V_{SW}} + {V_{PU}} \hfill \\ {\text{where, }}\left\{ \begin{gathered} {V_{SW}} = {V_{P - EX}} - {V_{PS}} \hfill \\ {V_{PU}} = {V_{dead}} + \frac{{{A_{mem}} \times \varepsilon }}{2} \hfill \\ \end{gathered} \right. \hfill \\ \end{gathered}$$

where, ${V}_{SW}$ and ${V}_{PU}$ are the swept and purge volumes, respectively. Further, subscripts $P-EX$ and $PS$ refer to the pressure exchanger and piston, sequentially. Also, ${A}_{mem}$ and $\varepsilon$ denote to the total membrane area and feed spacer thickness, respectively. Since in the BROU permeate and swept volumes are equal, the recovery ratio of the BROU is defined as the swept to the net volume^67^:

13 $$R{R_{BROU}} = \frac{{{V_{SW}}}}{{{V_{BROU,tot}}}}$$

In the batch reverse osmosis process, the permeate stream flows only in the pressurization mode. The permeate stream through the membrane is a function of the membrane area (${A}_{mem,BROU}$) and the water flux (${\varphi }_{W}$). The average permeate stream can be expressed by the following equation^66^:

14 $${Q_{PM,avg}} = R{R_{BROU}} \times \left( {{A_{mem,BROU}} \times {\phi_W}} \right)$$

Recirculation ratio is another vital parameter, which is defined as the ratio of the flow rate of the recirculation pump (${Q}_{RC}$) to the flow rate of the feed pump (${Q}_{feed}$):

15 $$\alpha = \frac{{{V_{SW}}}}{{{V_{PU}}}} = \frac{{{Q_{RC}}}}{{{Q_{feed}}}}$$

The specific power consumption of the BROU is based on the power consumed in the two phases of pressurization and purge-refill and auxiliary load. The auxiliary load is neglected due to its insignificant (compared to the other two components). Accordingly, the specific power consumption of the BROU can be formulated by the following equation^68^:

16 $${\overline P_{BROU}} = {\overline P_I} + {\overline P_{II}}$$

where, ${\overline{P} }_{I}$ and ${\overline{P} }_{II}$ are the specific power consumption of the pressurization and purge-refill phases, sequentially. The value of ${\overline{P} }_{I}$ is determined as^50^:

17 $${\overline P_I} = \frac{{{P_{VW,avg}}}}{{{\eta_{feed - P}}}} + \frac{{\alpha \times \Delta {P_{mem}}}}{{{\eta_{RC - P}}}}$$

where, ${P}_{VW,avg}$ and ${\Delta P}_{mem}$ are the volume-weighted average pressure and pressure drop in membrane module, respectively. Further, ${\eta }_{feed-P}$ and ${\eta }_{RC-P}$ refer to the feed and recirculation pumps efficiencies, respectively. Additionally, the value of ${\overline{P} }_{II}$ is calculated by:

18 $${\overline P_{II}} = \frac{{{P_{II,RC}}}}{{{\eta_{RC - P}}}} + \frac{{\Delta {P_{mem}}}}{{\alpha \times {\eta_{feed - P}}}}$$

The data required for the design of two BMEU and BROU processes are tabulated in Table 3.

**Table 3:** Data required for the design of two BMEU and BROU processes.

Parameter	Value	Parameter	Value
Temperature	25 °C	Pump efficiency	0.72
Gas constant	8.316 kJ/kmol.K	Membrane active area-BROU	33.9 m^2^
Current utilization	72%	Membrane length	964 mm
Recirculation ratio	2.9	Feed spacer thickness	0.864 mm
Cell thickness-BMEU	1 mm	Recovery ratio	74.5%
Osmotic water flow-Standalone	0.0010 L/h	Dead volume	6.85 L
Osmotic water flow-Combined	0.0513 L/h	Area resistance- bipolar membrane	1600 Ω/mm^2^
Water transport-Standalone	3.89 L/h	Area resistance-exchange membranes	1000 Ω/mm^2^
Water transport-Combined	3.95 L/h		

### Cost analysis

The cost analysis of the dairy wastewater treatment process proposed in this research is based on two main components (i.e., the costs of electrodialysis process with bipolar membrane and batch reverse osmosis process). The cost analysis of the BMEU is based on different process variables. The variable costs of the BMEU can be estimated based on two categories of predetermined costs (under the feed solution and product quality) and dependent costs (dependent on the membrane properties, working current density, stack geometry, etc.)^69^. In addition, the total production cost of the BMEU can be considered as the sum of fixed costs related to amortization of capital costs and operating costs (e.g., energy and maintenance costs):

19 $${{\text{C}}_{Pro,BMEU}} = {C_{fixed}} + {C_{operating}}$$

The total capital cost of a BMEU employed for the bases and acids production is a function of the needed membrane area (dependent on the plant capacity)^70^. Indeed, BMEU's investment cost is based on stack and peripheral equipment (e.g., control panels, monitoring, pumps, etc.) costs. The cost of the stack is proportional to the cost of the membrane, which is considered 1.5-fold the total cost of the membrane. In addition, the cost of peripheral equipment is considered equal to 50% of the cost of the stack. Note that, the cost of the membrane depends on the production scale and the membrane modules numbers. On the other hand, items such as electricity cost, membrane replacement cost, equipment depreciation, and other costs related to investment interest, labor, building construction, etc. represent the BMEU's maintenance costs. The assured and desirable lifetimes for membranes are reported to be about 1–2 years and 5 or more years, respectively. In the current research, the membrane lifespan is considered equal to 3 years. In addition, about 10–15% of the membranes must be replaced annually (here, 15%). Other costs related to the BMEU were assumed as 80% of the equipment depreciation cost. The BMEU's cost model is given in Table 4, which is based on previous reports^69–73^.

**Table 4:** The BMEU’s cost model.

Parameter	Value	Parameter	Value
BMEU-Stack cost	1.5-fold of membrane cost	Maintenance cost	10% of total capital cost
Ion exchange membrane cost	145 $/m^2^	Working times	24 h/day (330 day/year)
Bipolar membrane cost	1300 $/m^2^	Cost of energy	0.1 $/kWh
Peripheral equipment cost	0.5-fold of stack cost	Membrane lifetime-BMEU	3 years

The cost model of the BMEU based on membrane cost had been reported in some publications^69,70^. The cost data for the economic model of the BMEU are given in Table 5, which were extracted from the publications.

**Table 5:** Cost data for the economic model of the BROU.

Parameter	Value	Parameter	Value
Membrane lifetime-BROU	5 years	Piping cost	15% of equipment cost
Membrane replacement cost	20% of module cost	Peripheral equipment cost	10% of Capital cost
Unit membrane cost-BROU	22 $/m^2^		

The initial investment cost of the BROU is mainly based on the costs of membrane module and housing, high pressure and recirculation pumps, pressure exchanger and piping. Therefore, the equipment cost of the BROU can be formulated as follows^74^:

20 $${C_{eqp,BROU}} = {C_{HU}} + {C_{RC - P}} + {C_{HP - P}} + {C_{P - EX}}$$

where, $C$ is the cost. Further, subscripts $eqp$, $HU$ and $HP-P$ refer to the equipment, membrane housing and high pressure pump, respectively. The pumps costs are directly related to their working pressure ($P$) and flow rate ($Q$), which can be estimated as follows^74^:

21 $$\left\{ \begin{gathered} {C_{RC - P}} = 52 \times \left( {{P_{RC - P}} \times {Q_{RC}}} \right) \hfill \\ {C_{HP - P}} = 52 \times \left( {{P_{Peak}} \times {Q_{BROU}}} \right) \hfill \\ \end{gathered} \right.$$

where, ${P}_{RC-P}$ and ${P}_{Peak}$ are equivalent to the peak pressure of batch reverse osmosis cycle and the pressure drop across the membrane. In addition, the costs of the pressure exchanger and the membrane chamber are a function of the sweep volume purge volumes and peak pressure, which can be formulated by^71^:

22 $$\left\{ \begin{gathered} {C_{P - EX}} = 16 \times \left[ {{P_{Peak}} \times \exp \, \left( {4.3 \times {V_{SW}}} \right)} \right] \hfill \\ \hfill \\ {C_{HU}} = 16 \times \left[ {{P_{Peak}} \times \exp \, \left( {8.9 \times {V_{PU}}} \right)} \right] \hfill \\ \end{gathered} \right.$$

Finally, the cost of piping is assumed as 15% of the total cost of the equipment. Therefore, the capital expenditure (CAPEX) for the BROU can be expressed as follows:

23 $${\text{CAPE}}{{\text{X}}_{BROU}} = {C_{eqp,BROU}} \times 1.15$$

On the other hand, the membrane module cost can be described by the following equation, which is a function of the membrane area^75^:

24 $${C_{\bmod ule}} = {C_{mem}} \times {A_{BROU}}$$

Furthermore, the total operating expenditure (OPEX) of the BROU can be considered as the sum of membrane replacement cost, maintenance cost, and energy cost. The membrane replacement cost was assumed as 20% of the module cost and the maintenance cost was considered as 10% of the total CAPEX. Therefore, the total operating expenditure of the BROU can be formulated by the following equation^50^:

25 $$\begin{gathered} {\text{OPE}}{{\text{X}}_{BROU}} = {C_{O,\bmod ule}} + {C_{O\& M}} + {C_{EN}} \hfill \\ \, = \left( {{C_{\bmod ule}} \times 20\% } \right) + \left( {{\text{CAPEX}} \times 10\% } \right) + \left( {{{\Pr }_{en}} \times {{\overline P }_{BROU}}} \right) \hfill \\ \end{gathered}$$

**Figure 4:**
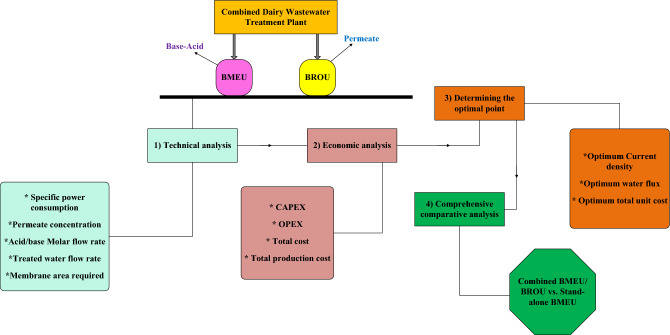
Structure of the design and analysis (flowchart of the steps in the manuscript) of the combined dairy wastewater treatment process under study.

The analysis method for the combined dairy wastewater treatment process is based on technical and financial analysis, as depicted in Fig. 4. Specifically, under technical analysis, parameters such as specific power consumption, permeate concentration, acid/base molar flow rate, treated water flow rate, required membrane area, optimal values of current density and water flux, etc. are calculated and analyzed. In addition, the financial analysis is based on the estimation of CAPEX and OPEX values, the total cost of the process, and finally the total production cost. Further, a comprehensive comparative analysis between the combined BMEU/BROU and the standalone BMEU is provided. Additionally, the assumptions applied in the design and analyses are listed in Table 6.

**Table 6:** Assumptions applied in the design and analyses.

No	Assumption
1	For both combined and stand-alone configurations, the diluted stream concentration is assumed to be the same
2	BMEU is based on the identical geometrical cells which are considered in co-current and co- velocity flows
3	The concentration is considered equivalent to the unit volume
4	It is assumed that the acid and base of BMEU can meet the quality requirements
5	The concentration potential is negligible compared to the resistances potentials
6	The variations effects in the solutions resistance are ignored
7	In both configurations, the water transfer rate is negligible due to electro-osmosis
8	The frictional pressure drop is not considered in the calculation (because it is negligible)
9	The costs related to the pre-treatment process (due to the lower share in the total cost) are not considered
10	Average amount of ~ 2.2 g/L of dissolved solids are available in the wastewater

## Results and discussion

The purpose of cost analysis is to achieve optimized cost modeling, which is developed under mathematical-cost modeling. Indeed, achieving the optimal cost model can identify solutions to achieve minimum capital and operating costs. In this section, the results related to both combined and stand-alone configurations are discussed and compared. Further, in this research, the optimal values of permeate flux and current density are discussed. In the studied BROU/BMEU combined configuration, the separation of dissolved salt and water during the wastewater treatment process of the dairy plant is done through BROU. Afterwards, the BROU reject flow is concentrated with dissolved salt and the mixture is directed to the BMEU inlet. Indeed, the design is to use the BROU before the BMEU to concentrate the feed flow. Accordingly, the concentrated feed flow is able to produce strong base-acid and reduce power consumption. Therefore, the properties of the reject flow can be the influencing factor on the performance of the wastewater treatment process.

**Figure 5:**
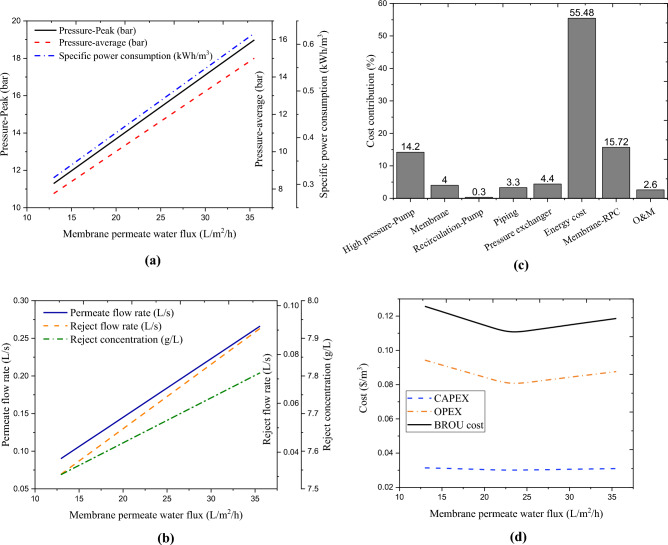
Effects of membrane permeate water flux on the technical and economic performances of the BROU in the studied wastewater treatment process: (**a**) peak and average pressures and specific power consumption, (**b**) permeate and reject flow rates and concentrate, (**c**) cost contributions, and (**d**) CAPEX, OPEX, and total cost.

Calculating the optimal permeate flux value (related to the BROU process) is one of the main objectives of this research. Membrane permeate water flux is a parameter that can vary during the operation of the wastewater treatment process. An enhancement in the permeate flux requires a raise in the pressure of the BROU. The effects of membrane permeate water flux on the technical and economic performances of the BROU in the studied wastewater treatment process are displayed in Fig. 5a–d. Figure 5a shows that to increase the permeate water flux, both peak and average pressures should increase linearly. The increase in pressure with the increase in permeate flux is due to the resistance of the pore friction. The findings indicated that raising the permeate flux by a certain percentage can enhance the resistance of the pore friction by almost the same percentage. In addition, the osmotic pressure and pressure drop in the membrane grow insignificantly with the increase of permeate flux. Finally, it is clear that increasing pressure means more power consumption. For this reason, the specific power consumption grows linearly with the increase of the permeate water flux. On the other hand, increasing the permeate water flux can improve the permeate flow rate (that is, the water output rate). In other words, rising the permeate flux means enhancing the feed injection into the process, which can also raise the rate of the reject flow. Figure 5b indicates that at higher permeate flux values, improved and higher values of permeate flow and reject flow reject can be achieved. This can also enhance the concentration of the reject stream. Since the reject flow is directed to the BMEU (as a feed), the improved concentration of reject flow can improve the performance of the BMEU. Therefore, as an important conclusion, improved performance in the studied dairy wastewater treatment process can be achieved at higher permeate water flux values.

The cost analysis of the BROU in terms of the permeate flux variations indicated that the increase in the permeate water flux can reduce the unit cost (UC) of the process. However, at higher values of permeate flux, equipment and operating costs of the BROU grow linearly. This is majorly due to the increased work (as well as equipment cost) of the high pressure pump and the consequent increase in energy cost. It was also found that the high pressure pump and energy have the largest shares, respectively, in the equipment and operating costs of the BROU, such that around 50% and 72% of the total equipment and operating costs of the BROU are related to these items, respectively (see Fig. 5c). In addition, in the BROU process, the OPEX term has a greater share in the total unit cost of water production compared to the CAPEX term. From Fig. 5d, it can be seen that the minimum (optimal) unit cost of water production under BROU process is 0.11 $/m^3^, which is obtained at a permeate water flux of nearly 23.6 L/m^3^/h. Therefore, point $$\left({\varphi }_{W}, UC\right)=\left(23.6, 0.11\right)$$ can be considered as an optimal point for the economic performance of the BROU. In such a situation, the return stream flow rate can be around 0.059 L/s.

**Figure 6:**
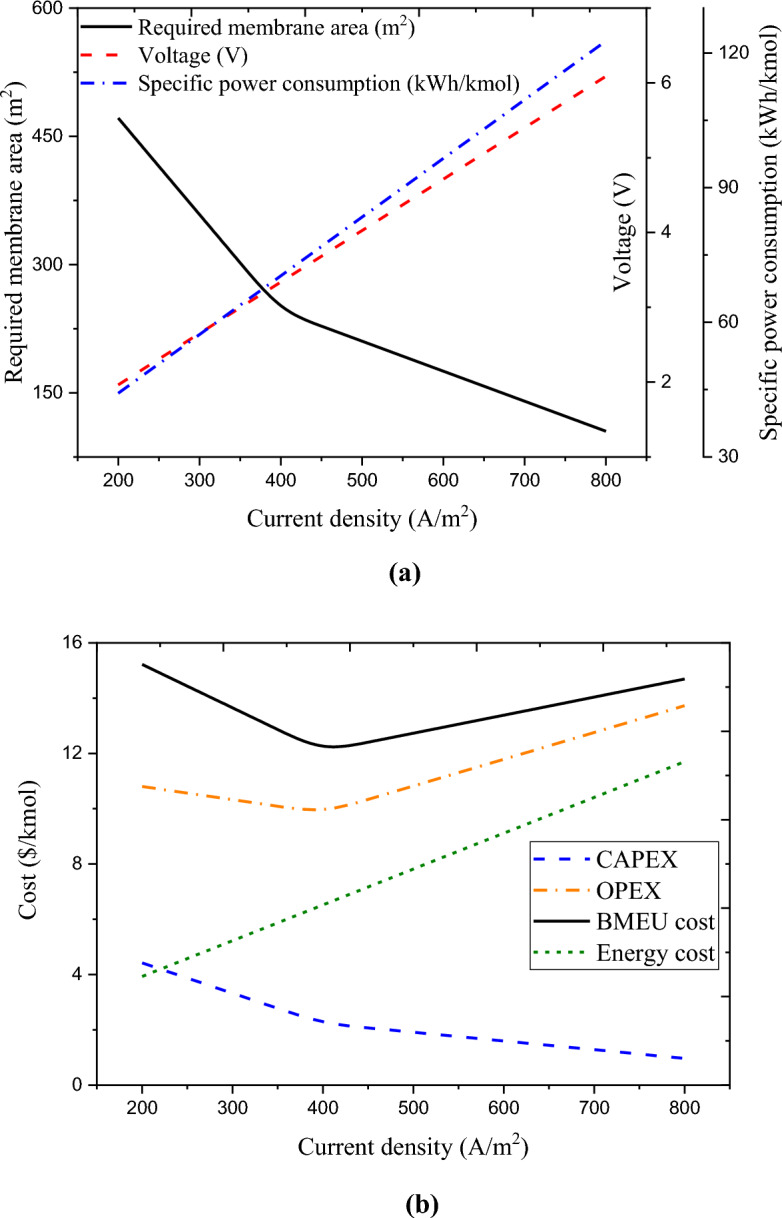
Effect of current density on the technical and economic performances of the BMEU in the studied wastewater treatment process: (**a**) required membrane area, cell voltage, and specific power consumption, and (**b**) CAPEX, OPEX, energy cost, and total cost.

Calculating the optimal value of current density (related to the BMEU process) is another objective of this research. The concentrated flow captured from the BROU is processed by the process carried out in the BMEU to produce the output flow at the same salinity as the input effluent (~ 2 g/L). This is equivalent to around 0.0076 mol/s of sodium chloride, which can be converted into base and acid by the transferred hydroxide ion and proton from the bipolar membrane. For the optimal design of the BMEU, it is necessary to determine the minimum product cost at an optimal current density value. Figure 6a,b depicts the effect of current density on the technical and economic performances of the BMEU in the studied wastewater treatment process. It can be seen that the investment cost (CAPEX value) of the BMEU decreases linearly with the increase in the current passing through the stack. The investment cost of the BMEU is a function of the stack cost, which directly depends on the membrane area. Due to assuming a constant rate of salt removal and based on Faraday's law, the current passing through the stack remains constant. While both applied voltage and the required area of the membrane are varied (due to maintaining the capacity of the plant constant). Although the membrane area decreases with the increase of the current passing through the stack, but due to draw necessary current, it is necessary to increase the applied voltage.

According to the plots, in lower values of the current density, the required area of the membrane decreases sharply, while in current densities higher than ~ 401 A/m^2^, the required area of the membrane decreases slower. For this reason, the investment cost of the BMEU experiences the same decreasing trend with increasing current density. However, the operating cost of the process first decreases with the increase in the current density passing through the stack, and after reaching the minimum point, it starts to increase. This is due to the reduction in the costs of membrane maintenance and replacement and the increase in the cost of electricity. With the increase in current density, all the costs related to the BMEU experience a decreasing trend, except for the energy cost. Because with the increase of the applied voltage, the specific power consumption and consequently the energy cost of the BMEU grow linearly. In fact, at current densities less than ~ 401 A/m^2^, the reductions in investment cost (CAPEX) and other operating costs (except energy cost) overcomes the enhancement in energy cost, which causes both the operating cost and the total cost of the BMEU process to drop. However, by passing currents higher than ~ 401 A/m^2^, the increasing trend of energy cost (due to the increase in specific power consumption) overcomes the decreasing trends of other items, which causes the increasing trends of operational cost and total cost. Therefore, it can be said that the membrane area the energy cost (due to the applied voltage) are two critical parameters in the total cost of the BMEU process. From Fig. 6c, it can be seen that the minimum (optimal) unit cost for the BMEU process is around 12.07 $/kmol, is achieved at ~ 401.6 A/m^2^. Therefore, point $$\left(j, UC\right)=\left(401.64, 12.072\right)$$ can be considered as an optimal point for the economic performance of the BMEU. In such a context, the specific power consumption can be around 66.31 kWh/kmoles (equivalent to producing 1.72 kWh/kg of sodium hydroxide). These results exhibit a good agreement compared to the experimental results reported by Culcasi et al.^76^.

### Performance of the combined BROU/BMEU wastewater treatment process

As discussed, the working permeate flux, which is one of the parameters related to the BROU, in addition to the BROU process, can affect the performance of the BMEU and, consequently, the whole studied wastewater treatment process. Accordingly, it is necessary to determine an optimal permeate flux to achieve the minimum total unit cost of the combined wastewater treatment process. In the combined wastewater treatment process, the flow and concentration rates of the reject stream directed to BMEU process are affected by the variations in the permeate water flux. Additionally, it was stated that the behavior of the BMEU process is completely affected by the working current density. Therefore, both the permeate water flux and the current density are the most critical parameters on the behavior of the combined dairy wastewater treatment process. Indeed, the overall unit cost of the combined process can be minimal in the optimal values of these parameters.

**Figure 7:**
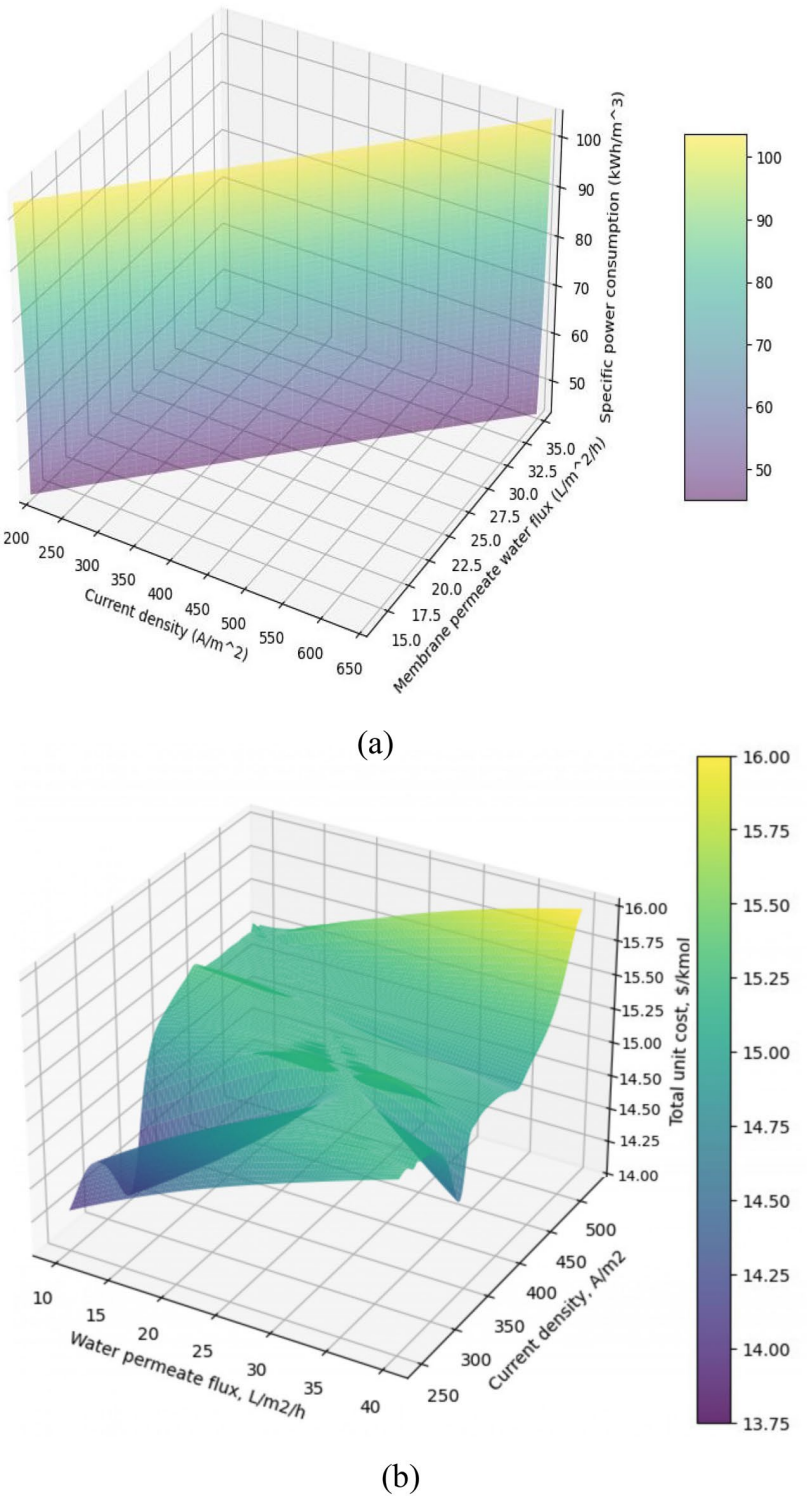
Simultaneous effects of permeate water flux and the current density on the specific power consumption and overall cost of the combined wastewater treatment process under study.

It is clear that the cost of BMEU will decrease with the increase of the permeate water flux (due to the improvement of the inflow concentration). The total cost of the studied combined wastewater treatment process is the sum of the costs of the BMEU and BROU processes. Indeed, the total cost of the combined process is determined by including the cost of the BROU in the cost of BMEU, which is determined by ratio of BROU cost to the annual base-acid outputs. Figure 7a,b displays the simultaneous effects of permeate water flux and the current density on the specific power consumption and overall cost of the combined wastewater treatment process under study. Since both parameters of membrane permeate water flux and current density increase the specific power consumption rate of the process, the simultaneous effects of infiltrated membrane permeate water flux and current density also lead to an upward trend in specific power consumption rate (see Fig. 7a). Further, according to the findings, the optimal points of $${\varphi }_{w}$$ and $$j$$ to achieve the minimum overall cost (~ 13.75 $/kmol) of the combined wastewater treatment process are equal to 25.53 L/m^3^/h and 401.64 A/m^2^, respectively. Furthermore, the ranges of $${\varphi }_{w}$$ and $$j$$ for the bottom plateau of the curve were observed as 16.7–29.3 L/m^3^/h and 343.7–466.5 A/m^2^, respectively. These can be considered as the operating ranges of the studied combined dairy wastewater treatment process. Because in these ranges, the overall cost of the process changes less than 1%.

**Figure 8:**
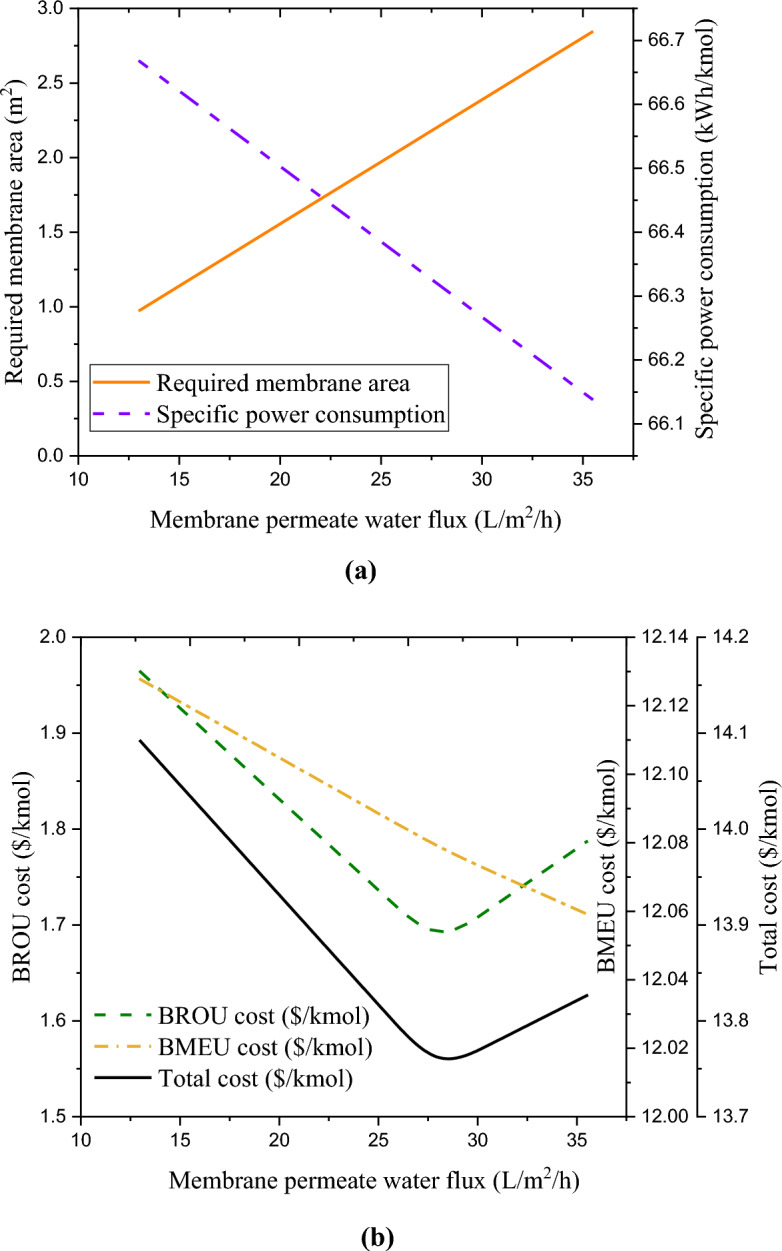
Effects of water flux on the technical and economic performances of the studied combined wastewater treatment process: (**a**) required membrane area and specific power consumption, and (**b**) BROU, BMEU, and total cost.

As discussed, the water flux affects the performance of both BROU and BMEU processes and consequently the overall performance of the combined process. Accordingly, the effects of water flux on the technical and economic performances of the studied combined wastewater treatment process are demonstrated in Fig. 8a,b. For any arbitrary value of permeate flux at the aforementioned range, the optimal current density value was around 401.6 A/m^2^. Based on this, the specific power consumption of the BMEU process in terms of different permeate fluxes under the optimal current density is plotted in Fig. 8a. Although the need for electric current enhances due to the improvement of the production rate in the BMEU, the applied voltage experiences a slight downward trend due to the lower electric resistance (caused by the increase in concentration). Since the growth rate of the electric current is higher than the reduction rate of the applied voltage, therefore, the consumption of electric energy experiences a significant upward trend. However, due to the increase in the feed volume flow rate, the specific power consumption decreases by around 1.18%.

On the other hand, due to the increase in the current passing through the stack, the required area of the membrane enhances significantly linearly with the increase in the permeate water flux; such that, by increasing the water flux from 13 to 37 L/m^3^/h, the required area of the membrane enhances by almost threefold. Interestingly, with the increase of the membrane area, the investment cost not only does not increase, but also experiences a slight decrease. This is due to the improvement of the production rate of the products, which nullifies the effect of the increase in the cost of the membrane. For this reason, the overall cost of the BMEU is reduced by 0.72% at the aforementioned range. The variations in the BROU cost also follow a similar trend.

**Figure 9:**
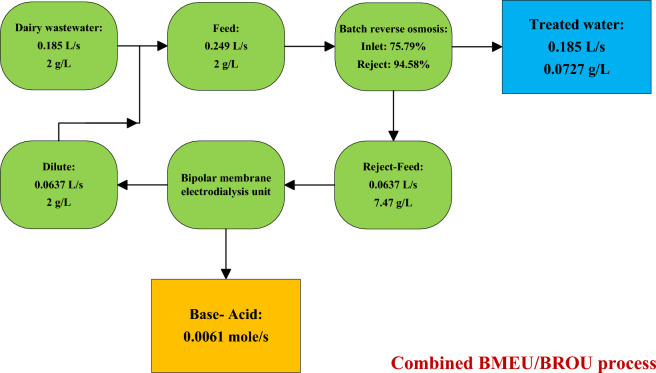
Block diagram of the process performance (concentration rate and volume flow) of the combined wastewater treatment process under optimal operating conditions.

Although the increase in the permeate water flux can greatly increase the cost of BROU process, due to the simultaneous improvement of the useful output rates, the cost of the BROU experiences a downward trend; such that by changing the water flux from 13 to 37 L/m^3^/h, the cost of BROU process declines by around 9%. As discussed earlier, a minimum point is observed for the costs of both BROU and BMEU processes. Therefore, the overall cost of the combined process of wastewater treatment also follows this variations trend (see Fig. 8b). Accordingly, the overall cost of the combined process of wastewater treatment is reduced by ~ 1.9% at the aforementioned range. The block diagram of the process performance (concentration rate and volume flow) of the combined wastewater treatment process under optimal operating conditions is illustrated in Fig. 9. It should be noted that in order to create a dynamic balance between the two BROU and BMEU processes, it is necessary to use an intermediate tank.

### Performance of the stand-alone BMEU wastewater treatment process

**Figure 10:**
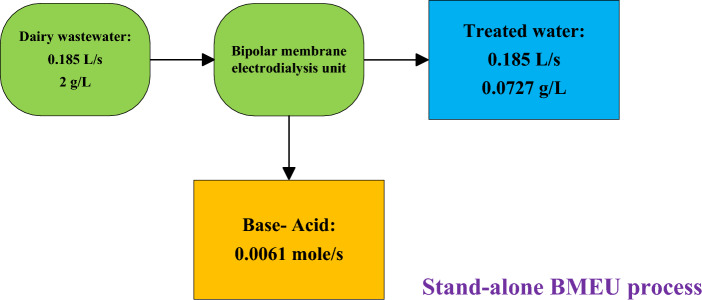
Block diagram of the process performance of the stand-alone wastewater treatment process.

In the second proposed configuration, the dairy wastewater is directly treated by the BMEU process and in addition to producing water; it can also produce base and acid. Note that, the analysis process of the second proposed configuration is also based on the mathematical simulation and modeling. To compare the two proposed configurations, the input and output data of the combined process are also applied to the stand-alone process. Indeed, similar to the combined configuration, the input flow rate to the process is set at 0.185 L/s, which is capable of producing salt and treated water at the rates of 0.0061 mol/s and 0.185 L/s, respectively. Figure 10 depicts the block diagram of the process performance of the stand-alone wastewater treatment process. In the stand-alone process, wastewater treatment should be performed at a salinity of less than 2 g per liter. In addition, similarly, the concentration rate of the treated water is assumed to be 0.072 g/L.

**Figure 11:**
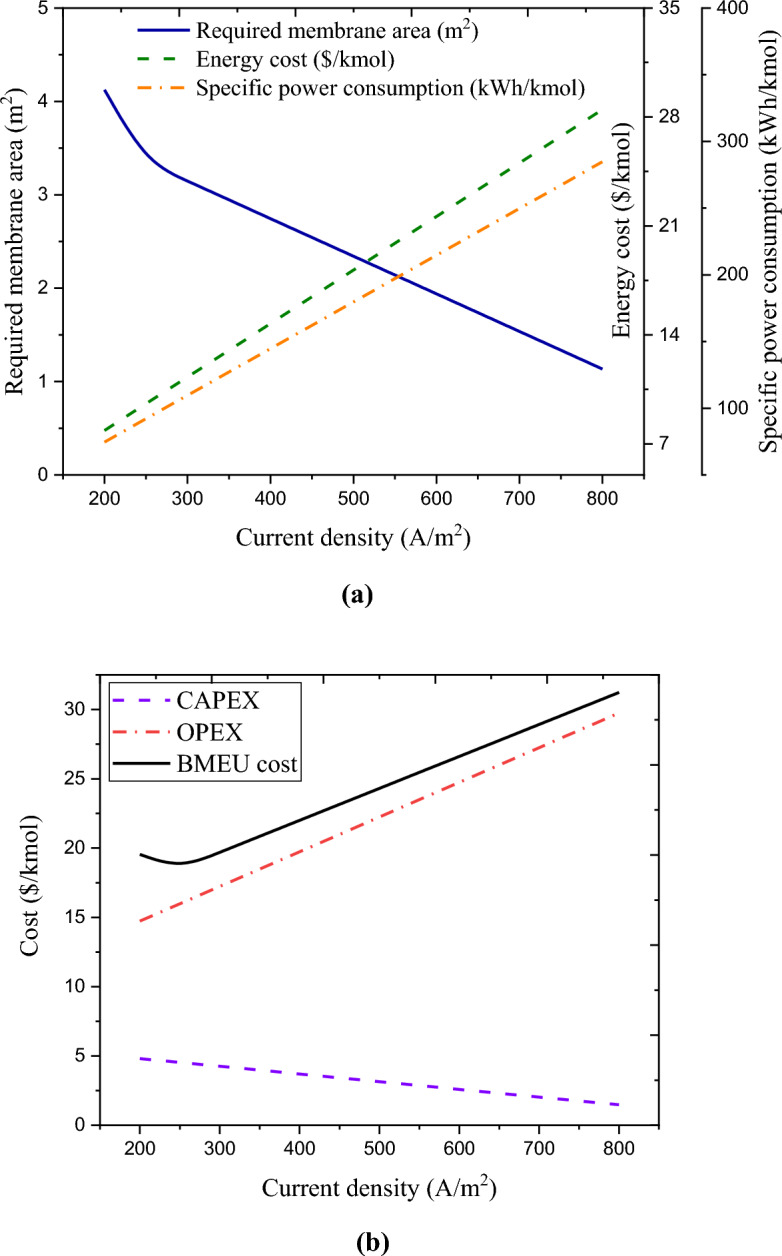
Effect of current density on the technical and economic performances of the stand-alone wastewater treatment process: (**a**) required membrane area, energy cost, and specific power consumption, and (**b**) CAPEX, OPEX, and total cost.

In both configurations, the water transfer rate is negligible due to electro-osmosis. According to the calculations, the water transfer rate in both configurations is around 0.6%, which is ignored for this reason. Figure 11a,b displays the effect of current density on the technical and economic performances of the stand-alone wastewater treatment process. Due to different operating conditions, the technical and economic performances of the process are different from the first configuration. It can be seen that the minimum (optimal) total cost for the stand-alone wastewater treatment process is about 18.55 $/kmol, is determined at 250.4 A/m^2^. Therefore, point $$\left(j, {C}_{tot}\right)=\left(250.4, 18.54\right)$$ can be considered as an optimal point for the economic performance of the stand-alone wastewater treatment process.

The reason for the increase in the overall cost of the stand-alone configuration with the increase in current density is due to the linear growth of the specific power consumption of the process. All costs of the stand-alone configuration (except energy cost) experience a hyperbolic downward trend with raising current density (due to the reduction of equipment costs). Figure 11 also exhibits that the required area of the membrane decreases until the optimal point of the current density with high intensity and then the decreasing rate becomes more balanced. Energy cost and specific power consumption also grow with a similar trend.

**Figure 12:**
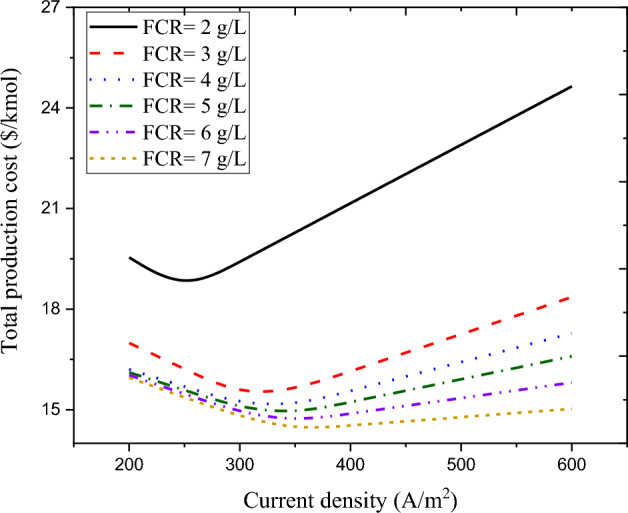
Influence of current density on overall product cost of the wastewater treatment plant at different FCR values.

Feed concentration rate (FCR) is another parameter that affects the economic performance of the wastewater treatment process. Figure 12 displays the influence of current density on overall product cost of the wastewater treatment plant at different FCR values. Increasing the feed concentration rate can reduce the amount of applied voltage due to the reduction of electrical resistance. This in turn reduces specific power consumption and energy cost. Variations in the feed concentration rate do not affect the capital cost of the wastewater treatment process due to the constant membrane area. Therefore, due to the reduction of the operating cost (by raising the feed concentration rate), the overall product cost of the wastewater treatment process is also reduced. The optimum points of current density and total product cost are different for different feed concentration rates. It should be noted that in this evaluation, the salt removal rate is considered constant for different feed concentration rates. By increasing the feed concentration rate from 2 to 7 g/L, the optimum value of the total product cost can be declined by around 22.7%. However, the optimum point of current density increases by about 79 A/m^2^. Therefore, evaluating the overall product cost by simultaneously considering the effects of current density and feed concentration rate can lead to a suitable optimal point for cost analysis of the studied wastewater treatment process. Accordingly, point $\left(j, FCR, {C}_{tot}\right)=\left(328.9, 7, 14.37\right)$ can be considered as an optimal point for the economic performance of the studied wastewater treatment process.

### Comparison of two stand-alone and combined configurations

The technical and economic comparisons of two different configurations proposed for the wastewater treatment process can be a good guide for engineers and stakeholders in deciding to adopt the appropriate configuration. In this regard, in this subsection, a comprehensive comparison of the performance of both stand-alone and hybrid configurations is presented. The two configurations provide different technical and economic performances due to the different input data of the BMEU process in stand-alone and combined configurations. For stand-alone and combined configurations, the rates of feed volume flow of the BMEU are 0.185 and 0.063 L/s, respectively. Moreover, for stand-alone and combined configurations, the feed concentration rates of the BMEU are 2 and 7.6 g/L, respectively. According to the calculations, the optimal current densities for two stand-alone and combined configurations were obtained as 250.4 and 401.6 A/m^2^, respectively. Further, the unit costs of the BMEU for two stand-alone and combined configurations were determined as 18.54 and 12.072 $/kmol, respectively.

**Figure 13:**
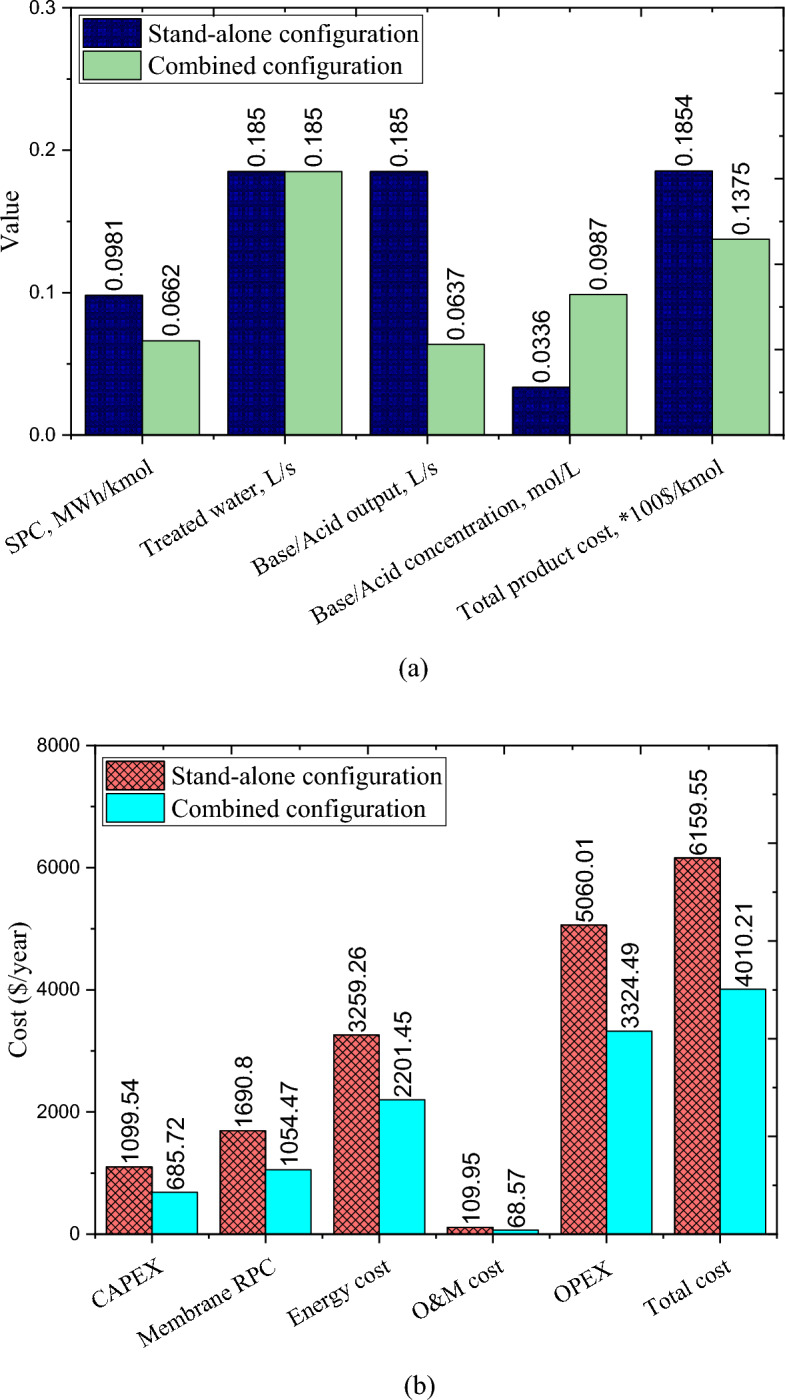
Results of the comparison of two stand-alone and combined wastewater treatment configurations: (**a**) overall comparison and (**b**) cost comparison.

The results of the comparison of two stand-alone and combined wastewater treatment configurations are displayed in Fig. 13a,b. The treated water rate and annual production of the base and acid are the same in both configurations. However, the specific power consumption and required area of the membrane in the stand-alone configuration are approximately 47.9% and 60.3%, respectively, higher than that of the combined configuration. Note that, although the volume flow rate of base and acid produced in the combined configuration is lower than that in the stand-alone configuration, the high concentration rate of base and acid produced in the combined configuration makes the annual output rate of these products the same in both configurations. Figure 13b shows that the total cost of the stand-alone configuration is approximately 53.6% higher compared to the combined configuration. This is due to the high capital and operating costs of the stand-alone configuration compared to the combined configuration. The energy cost in the stand-alone configuration is around 48% higher than the combined configuration.

Other cost items are more in stand-alone configuration than in combined configuration. In addition, according to the findings of the research, the total cost of production in the combined configuration has been reduced by approximately 26% compared to the stand-alone configuration. Therefore, although both stand-alone and hybrid configurations provide almost similar technical performance, the combined configuration can address significantly superior economic performance compared to the stand-alone configuration. Accordingly, increasing the feed concentration rate using the batch reverse osmosis process (before the electrodialysis process with bipolar membrane) for the dairy wastewater treatment process can be a fruitful and ideal solution from an economic point of view. Due to the reutilize of wastewater treatment products in the dairy plant, the proposed plan can deal with the concept of circular economy and wastewater management.

### Comparison of results

As mentioned, the total production cost in the studied combined dairy wastewater treatment process can be around 13.75 $/kmol (base/acid). To verify the results and highlight the superiority of the economic performance of the proposed process, a comparison with similar reported technologies is presented. Davis et al.^69^ employed BMEU for base/acid production from dilute salt solutions that were obtained under a reverse osmosis process. They reported the total production costs of acid (Hydrochloric acid) and base (Sodium hydroxide) to be 37 and 21 $/kmol, respectively. Tang et al.^41^ employed BMEU to treat the concentrated brine obtained during reverse osmosis process for recover valuable resources and generate base-acid. They reported the total production cost of base (Sodium hydroxide) to be and 57.6 $/kmol. Sun et al.^77^ developed a Sulfanilic acid wastewater desalination strategy under BMEU to separate Sulfanilic acid and Sodium chloride from wastewater and convert to Hydrochloric acid and Sodium hydroxide. They reported the total production costs of acid (Hydrochloric acid) and base (Sodium hydroxide) to be 61.3 and 60.8 $/kmol, respectively. Culcasi et al.^78^ developed a fully integrated multi-scale model for the potential of BMEU. They reported the total production cost of base (Sodium hydroxide) to be and 14.4 $/kmol. Therefore, the proposed combined process for the treatment and recovery of dairy wastewater can address superior economic performance and competitive with other reported technologies. It can deal with the concept of circular economy and wastewater management.

## Conclusions

Industrial wastewater treatment can jointly address energy and environmental issues. This article aimed to develop and evaluate a combined BMEU/ BROU process for the recovery of chemicals and water from the dairy wastewater plant. In the proposed combined process, it was possible to simultaneously produce water and base-acid from the final dairy effluent. The technical and cost analyses of the combined wastewater treatment process were comprehensively discussed and investigated. In addition, a comprehensive comparative analysis on the performances of two combined and stand-alone BMEU configurations were developed. The proposed combined technology for dairy factory wastewater treatment was designed on a new structure and configuration that can address superior cost analysis compared to similar technologies. From the outcomes, point (j,FCR,C_tot) = (328.9,7,14.37) can be considered as an optimal point for the economic performance of the studied wastewater treatment process. Other main observation as:

- The improved performance in the studied dairy wastewater treatment process can be achieved at higher permeate water flux values. Further, the increase in the permeate water flux can reduce the unit cost of the process.
- The high pressure pump and energy have the largest shares, respectively, in the equipment and operating costs of the BROU, such that around 50% and 72% of the total equipment and operating costs of the BROU are related to these items, respectively.
- The membrane area and lifetime as well as the energy cost (due to the applied voltage) are two critical parameters in the BMEU cost. The minimum (optimal) unit cost for the BMEU process is around 12.07 $/kmol, is determined at a ~ 401.6 A/m^2^.
- The optimal points of $${\varphi }_{w}$$ and $$j$$ to achieve the minimum overall cost (~ 13.75 $/kmol) of the combined wastewater treatment process are equal to 25.53 L/m^3^/h and 401.64 A/m^2^, respectively. Furthermore, the ranges of $${\varphi }_{w}$$ and $$j$$ for the bottom plateau of the curve were observed as 16.7–29.3 L/m^3^/h and 343.7–466.5 A/m^2^, respectively. These can be considered as the operating ranges of the studied combined dairy wastewater treatment process. Because in these ranges, the overall cost of the process changes less than 1%.
- The total cost of production in the combined configuration has been reduced by approximately 26% compared to the stand-alone configuration. Therefore, although both stand-alone and hybrid configurations provide almost similar technical performance, the combined configuration can address significantly superior economic performance compared to the stand-alone configuration.

Increasing the feed concentration rate using the batch reverse osmosis process (before the electrodialysis process with bipolar membrane) for the dairy wastewater treatment process can be a fruitful and ideal solution from an economic point of view. Due to the reutilize of wastewater treatment products in the dairy plant, the proposed plan can deal with the concept of circular economy and wastewater management. The planned combined process for the treatment and recovery of dairy wastewater can address superior economic performance and competitive with other reported technologies. However, it is recommended to develop environmental life cycle analysis in future works. The limitation of the research is the assumption that the engineering requirements are met by the accuracy of the developed model. In addition, another limitation of the research is the assumption of average pressure for BROU with unsteady state operation. Although in the results and discussion section, the outcomes of the research were compared with similar reports in the literature and the accuracy of the model was confirmed, however, it is recommended that a small-scale experimental work be considered. Because experimental work compared to modeling is faced with hundreds of predicted and unpredicted parameters that can affect the accuracy of modeling.

## Data Availability

The datasets used and/or analysed during the current study available from the corresponding author on reasonable request.

## List of symbols

A: Area (m^2^)
AR: Area resistance
C: Cost ($)
CP: Concentration
F: Faraday constant
H: Equivalent conductivity
I: Current (A)
j: Current density (A/m^2^)
L: Length (cm)
N: Cell pair numbers
P: Pressure (bar)
$$\overline{\mathbf{P} }$$: Specific power consumption (kWh/kmol)
ΔP: Pressure drop
Q_f_: Feed flow rate (L/s)
R: Gas constant (J/mol.K)
RR: Recovery ratio
T: Temperature (^°^C)
U_I_: Current utilization
V: Voltage (V)
ΔV: Voltage drop (V)
W: Cell width

## Greek symbols

α: Recirculation ratio
ε: Cell thickness
η: Efficiency (%)
φ_w_: Water flux (L/m^2^/h)

## Subscripts

a: Acid
amem: Anion-exchange membrane
avg: Average
b: Base
bm: Bipolar membrane
cmem: Cation-exchange membrane
en: Energy
eqp: Equipment
HP: High-pressure
HU: Housing
in: Inlet
mem: Membrane
O: Operating
out: Outlet
P: Pump
P-EX: Pressure exchanger
PS: Piston
PU: Purge
RC: Recirculation
s: Salt
SW: Swept
tot: Total
tr: Transition region
VW: Volume-weighted

## Abbreviations

BMEU: Bipolar membrane electrodialysis unit
BROU: Batch reverse osmosis unit
CAPEX: Capital expenditure
FCR: Feed concentration rate
HCL: Hydrochloric acid
NaOH: Sodium hydroxide
OPEX: Operating expenditure
UC: Unit cost
